# Digital Breast Tomosynthesis: Towards Dose Reduction through Image Quality Improvement

**DOI:** 10.3390/jimaging9060119

**Published:** 2023-06-11

**Authors:** Ana M. Mota, João Mendes, Nuno Matela

**Affiliations:** 1 Faculdade de Ciências, Instituto de Biofísica e Engenharia Biomédica, Universidade de Lisboa, 1749-016 Lisboa, Portugal; jpmendes@fc.ul.pt (J.M.); nmatela@fc.ul.pt (N.M.); 2 Faculdade de Ciências, LASIGE, Universidade de Lisboa, 1749-016 Lisboa, Portugal

**Keywords:** digital breast tomosynthesis, total variation minimization, image quality, dose reduction

## Abstract

Currently, breast cancer is the most commonly diagnosed type of cancer worldwide. Digital Breast Tomosynthesis (DBT) has been widely accepted as a stand-alone modality to replace Digital Mammography, particularly in denser breasts. However, the image quality improvement provided by DBT is accompanied by an increase in the radiation dose for the patient. Here, a method based on 2D Total Variation (2D TV) minimization to improve image quality without the need to increase the dose was proposed. Two phantoms were used to acquire data at different dose ranges (0.88–2.19 mGy for Gammex 156 and 0.65–1.71 mGy for our phantom). A 2D TV minimization filter was applied to the data, and the image quality was assessed through contrast-to-noise ratio (CNR) and the detectability index of lesions before and after filtering. The results showed a decrease in 2D TV values after filtering, with variations of up to 31%, increasing image quality. The increase in CNR values after filtering showed that it is possible to use lower doses (−26%, on average) without compromising on image quality. The detectability index had substantial increases (up to 14%), especially in smaller lesions. So, not only did the proposed approach allow for the enhancement of image quality without increasing the dose, but it also improved the chances of detecting small lesions that could be overlooked.

## 1. Introduction

Recent data shows that breast cancer (BC) is nowadays responsible for one in eight new cancer diagnoses, making it the most frequently diagnosed cancer worldwide [1]. The increase observed in BC incidence rates over the last few years [2] might indicate that in the future a continuation of growth in the number of newly diagnosed BC cases is going to be observed. In fact, the number of yearly diagnosed cases is expected to be more than 3 million by 2040—this represents an increase in comparison to the number of BC cases diagnosed in 2020, which was 2.3 million [1].

Screening routines for BC are usually performed via digital mammography (DM), and it is acknowledged how these programs have contributed to a generalized decrease in BC death rates [3]. Nonetheless, its 2D acquisition nature originates from tissue overlap, which can result in tumor obscuring and/or the creation of false lesions [4]. This means that during their lifetime, about 50% of women in the United States of America will receive a false-positive result—which can lead to a high anxiety state and unnecessary follow-up exams [5].

Digital Breast Tomosynthesis (DBT), an imaging technique that consists of a 3D volume data resulting from a reconstruction of several low-dose X-ray images acquired at different angles, presents itself as a possible solution to overcome the issue of overlapping tissue [6]. As a matter of fact, studies point out that the combined use of DBT and DM increased the cancer detection rate by 40% and decreased false-positive rates by 15% in comparison to the single use of DM [7], particularly with dense breasts [8]. Currently, by including synthetic mammography generated from DBT data, DBT alone is accepted as a stand-alone modality to replace DM [9,10,11,12,13,14].

As DBT is composed of several X-ray images, there might be some concerns related to the total radiation dose given to the patient, in comparison to that associated with classic DM. The variation in the radiation dose is very well established in the field, and there are several works that report an increase when DBT is used instead of mammography (or in combination with it). Specifically, the use of DBT alongside mammography can lead to double the radiation exposure than just for mammography [15]. A study [16] found a mean increase of 34% and 46% in entrance skin air kerma and average glandular dose, respectively. A dose 13% higher that was used for DBT in comparison to mammography was found in a study for a breast thickness that was “close to average size breast”. Finally, a group of researchers found that there is a statistically significant increase in the dose given to the patients when performing the transition from DM to DBT. The average value for this increase was 37% with the maximum value going up to 75% [17]. Given the analyzed results, it is noted that the advantages driven by the use of DBT are accompanied by an increase in the absolute dose given to the patient. Furthermore, since DBT is obtained through several low-dose acquisitions, the reconstructed volume will bear a considerable amount of noise. Therefore, it is important to understand how to benefit from this “free-of-overlap” technique without the need to increase the dose to achieve good image quality. As DBT is being increasingly used in breast cancer detection, it is mandatory to ensure that the dose for the patients in DBT exams remains as low as possible. For that reason, the optimization of the radiation dose is an open issue which has been continuously explored. The literature presents a variety of approaches to try to achieve a lower dose while maintaining the desired image quality. Many of the studies were conducted using simulations of dose reduction through the injection of quantum noise in clinical images [18,19], deep-learning-based image processing techniques for radiation dose reduction in DBT [20,21], post-processing filters to low-dose projections [22], or new iterative reconstruction methods [23,24]. However, there is no consensus on its use and the performed analyses are limited.

Total variation (TV) minimization—a method that aims to reduce the amount of pixel intensity variation across the image—has shown promising results in the task of removing noise while preserving the image’s edges [25]. The results obtained with TV methods are dependent both on the Lagrange multiplier (λ) that controls the compromise between noise removal and edge preservation, and on an appropriate choice of a noise model—which needs to be close to the distribution seen in the images from where the noise is going to be removed [26]. Common methods using TV for filtering assume that the image has a random Gaussian noise distribution, but this fact might not be the case for every case of medical imaging data [27]. This research focuses on the TV minimization problem using Poisson noise distribution [28].

TV minimization techniques have produced positive results in several medical imaging modalities. For example, it has been used in the reconstruction of low-dose CT [29] and MRI [30]. Given that, the use of this technique for the diminishing of noise in DBT data with edge preservation might be advantageous. In fact, several works have already focused on the use of TV applied to DBT. One of them aimed to compare three different reconstruction techniques: algebraic reconstruction and a combination of it with both 2D and 3D TV. The obtained results showed that the use of 3D TV helped to a achieve a faster reconstruction with the presence of less artifacts [31]. A further development of new versions of TV methods for DBT applications show that it is yet possible to improve image quality after reconstruction [32,33].

Previous work from our group in this area resulted in the proposal of a TV filter to improve the quality of DBT images. Actually, when the proposed method was applied to a phantom, the Signal Difference-to-Noise Ratio (SDNR) achieved an increase of up to 5.44% and to 8.32% when applied before and after reconstruction, respectively. When the filter was applied to real-world clinical images, the improvement achieved on SDNR was 24.39% [26].

All the analyzed results, with a special focus on the work developed by our team, show the potential that TV has in the field of noise removal on DBT images while maintaining the edges unaffected.

In this paper, this 2D TV minimization problem is approached to study the possibility for reducing radiation dose in DBT. The image quality assessments are performed using two important and distinct metrics: Contrast-to-noise ratio (CNR) and detectability index. The performance of the filter is studied on the reconstruction data of two different phantoms, acquired for several radiation doses.

## 2. Materials and Methods

### 2.1. Data Acquisition and Reconstruction

**Figure 1:**
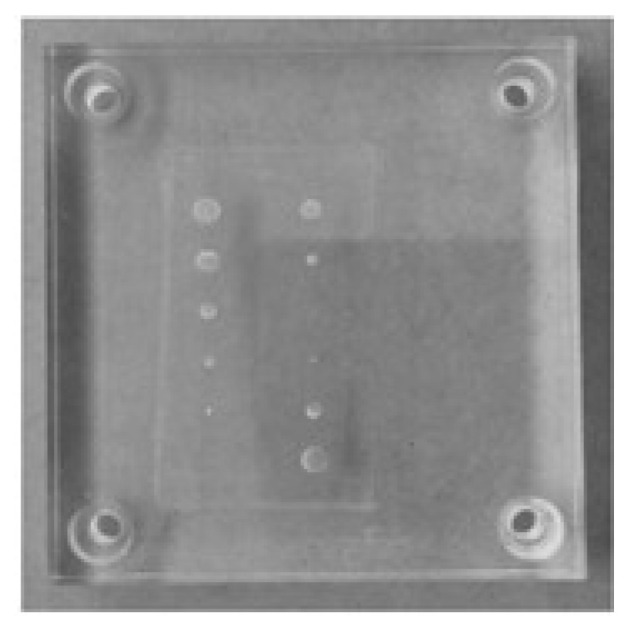
Acrylic phantom simulating breast tissue and lesions of high attenuation (aluminum disks of different diameters and 1 mm thickness). Diameters of the disks of the first column (**top to bottom**): 5.0 mm, 4.0 mm, 3.0 mm, 2.0 mm, 1.0 mm, and 0.5 mm, respectively; Diameter of the disks of the second column (**top to bottom**): 4.0 mm, 2.0 mm, 0.5 mm, 1.0 mm, 3.0 mm, and 5.0 mm, respectively.

In this work, two phantoms were used. The first was the Mammographic Accreditation Phantom Gammex 156 (Gammex, Inc., Middleton, WI, USA) and the second was an acrylic phantom that mimics the breast tissue, made by us (Figure 1). The two were acquired to study different imaging features. The first because it contains soft tissue lesions with a contrast very similar to the surrounding tissue. The second because it simulates high-density lesions of different sizes with high contrast in relation to the background (Figure 1). Both were acquired with a Siemens MAMMOMAT Inspiration system (Siemens AG, Healthcare sector, Erlangen, Germany) in a clinical facility (Hospital da Luz S.A., Lisbon, Portugal).

The acquisition parameters which directly affect image quality are the peak kilo-voltage output of the X-ray generator used (kVp) and the exposure value (expressed in milliampere-second, mAs). The first defines the penetration level of the X-rays, controlling the contrast of the image, and the second is responsible for the tube current, controlling the noise. Of the two, exposure (mAs) is the one that relates to a greater degree to the patient dose. In this work, for each phantom, several acquisitions were obtained with different mAs values (and consequently, different dose values), keeping constant the kVp. The acquisition parameters used have been carefully studied to match those observed in the clinical data, and they are described in Table 1 for both phantoms. The exposure expressed in mAs was calculated from the exposure time (millisecond, msec) and the X-ray tube current (milliampere, mA), and the dose is the average breast dose estimated in miligray (mGy) during the acquisition.

The acquired data were reconstructed with voxel sizes of 0.085 × 0.085 × 1.0 mm${}^{3}$ using the manufacturer algorithm, which uses Filtered Back Projection (FBP) with post-processing techniques. The methods under study were implemented using MATLAB R2020b and run on a computer Intel(R) Core(TM) i7-7700K CPU @ 4.20GHz.

### 2.2. Formulation of the TV Minimization Problem

The filter developed by our team in [26] was applied in this study. This filter is based on the 2D total variation (TV) of the image, calculated through Equation (1), where $u_{i,j}$ is the intensity value of pixel (i,j), with i=1,…,m, j=1,…,n and mn being the image dimensions. $\Delta_{x}$ and $\Delta_{y}$ are the discretizations of the horizontal and vertical derivatives, respectively.

(1) $$TV\left(u\right)=\sum_{i=1}^{m}\sum_{j=1}^{m}\sqrt{\left(\Delta_{x}u_{i,j}^{2}\right)+\left(\Delta_{y}u_{i,j}^{2}\right)}$$

Through an effective method of minimizing the 2D TV of the pixel intensity in the image, remarkable levels of noise reduction were achieved without producing additional blur. In this way, after these good results and through noise reduction with these algorithms, the present work was carried out in order to study the possibility of dose reduction in DBT while preserving the same image quality. The 2D TV minimization algorithm’s performance directly depends on a regularization parameter (the Lagrange multiplier, λ). λ allows for the control of the weight between the regularization vs. fidelity terms in the filter equation [26], and there is only one λ value for which the minimum 2D TV is obtained, while maintaining the fidelity of the data. So, before applying the filter, this optimal λ value was found for each phantom and each acquisition dose of Table 1.

### 2.3. Image Analysis

Image quality assessments were carried out by measuring the contrast-to-noise ratio (CNR) and the detectability index in the original and filtered data.

**Figure 2:**
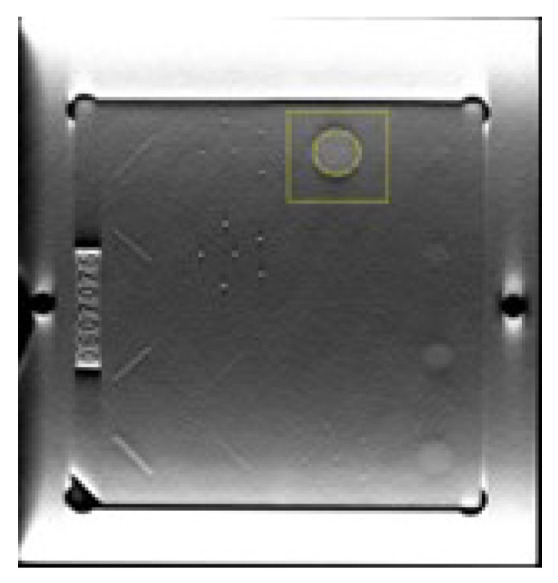
ROIs used to calculate CNR using the Gammex 156 phantom. Circular ROI over the 2 mm lesion-like mass and square background ROI centered in the lesion, excluding all voxels corresponding to the lesion.

For CNR, a region of interest (ROI) over the 2 mm lesion-like mass and another over the background were drawn at the in-focus slice. The background ROI was drawn as a square centered in the lesion, excluding all voxels corresponding to the lesion (Figure 2). CNR was calculated with Equation (2), where $\mu_{L}$ and $\mu_{B}$ stand for the mean pixel values in ROI over the lesion and background, respectively, and $\sigma_{B}$ stands for standard deviation in background ROI.

(2) $$CNR=\frac{\mu_{L}-\mu_{B}}{\sigma_{B}}$$

As CNR can be measured relatively quickly and easily, it is a traditional and widely used image quality metric for the determination of noise reduction. However, to characterize or to compare image quality between imaging conditions with variable noise magnitude or resolution (for example, for different reconstruction algorithms), task-based image quality metrics such as the detectability index (*d*′) should be used (Equation (3) [34]. In this work, both metrics were calculated so that the methods could be reproducible and the results broadly comparable.

(3) $${d^{\prime }}^{2}=\frac{\left[\int \int \right|W_{task}\left(u,v\right){{|}^{2}.TTF^{2}\left(u,v\right)dudv]}^{2}}{\int \int |W_{task}{\left(u,v\right)|}^{2}.TTF^{2}\left(u,v\right).NPS\left(u,v\right)dudv}$$

**Figure 3:**
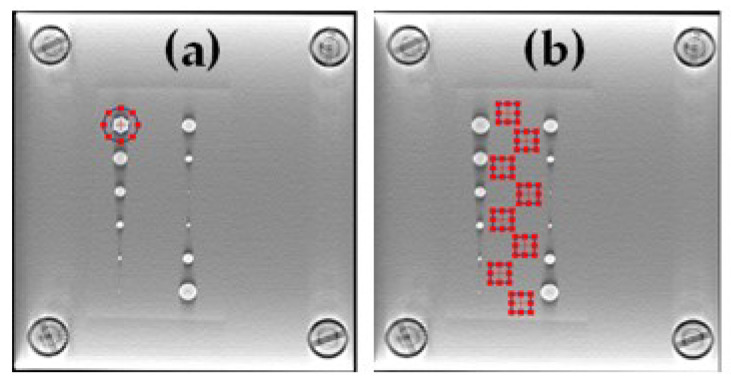
(**a**) ROI used to compute the task-based transfer function (TTF). (**b**) ROIs used for the noise power spectrum (NPS) assessment.

In Equation (3), TTF is the task transfer function and is a measurement that is analogous to the modulation transfer function for a non-linear imaging system. Here, the in-plane TTF was measured based on a circular ROI around the 5.0 mm disk available in our phantom (Figure 3a). From this ROI, it is possible to identify the center of the disk, and then to calculate the distance of each pixel in the ROI from the center, which will result in the edge spread function (ESF). The line-spread function (LSF) was obtained using the derivative of a smooth ESF, and the TTF was computed from the normalized Fourier transformation of the LSF. The noise power spectrum (NPS) was computed by placing eight square ROIs in the uniform section of the phantom (Figure 3b).

**Figure 4:**
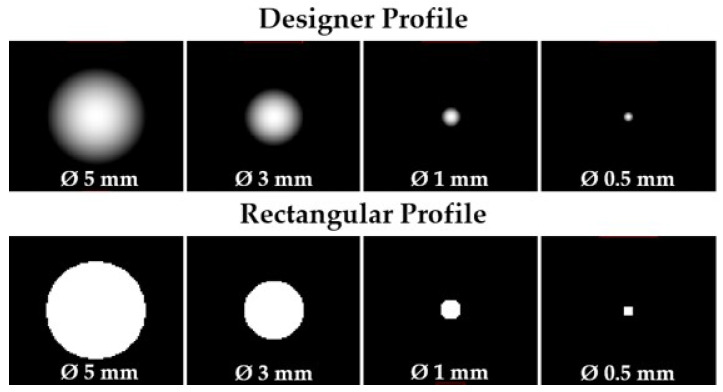
The **top row** shows the synthesized signals of four different sizes to be detected with a designer contrast profile, and the **bottom row**, with a rectangular contrast profile. The Fourier transform of such a signal is the task function, $W_{task}$.

$W_{task}$ is the task function and it is the Fourier transform of the signal to be detected. Signals of four sizes (5.0 mm, 3.0 mm, 1.0 mm, and 0.5 mm) were used with a designer contrast profile and a rectangular contrast profile (Figure 4). Those sizes and profiles were chosen to englobe the diversity of lesions present in DBT (from large masses with a low sharpness of the edge-designer profile, to small, well-defined microcalcifications—rectangular profile). TTF, NPS, Wtask, and the detectability index (*d*′), based on the non-prewhitening (NPW) matched filter model, were calculated using imQuest software (imQuest, Duke University, Durham, NC, USA). The methods used by the software and followed in this work are part of the American Association of Physicists in Medicine task group report, TG-233 [34].

## 3. Results

The results were obtained for an in-focus slice for each phantom (slice number 36 for Gammex 156, and slice number 42 for our phantom). Each application of the filter was performed in approximately 0.11 s for both Gammex and our phantom.

Table 2 shows the 2D TV values for the original data and for the data after the filtering routine was applied. The data evaluated in this table were obtained with the Gammex 156 phantom at four different dose values (with the optimum λ being found for each dose). Furthermore, the variations between the TV values before and after filtering are also present in Table 2.

**Figure 5:**
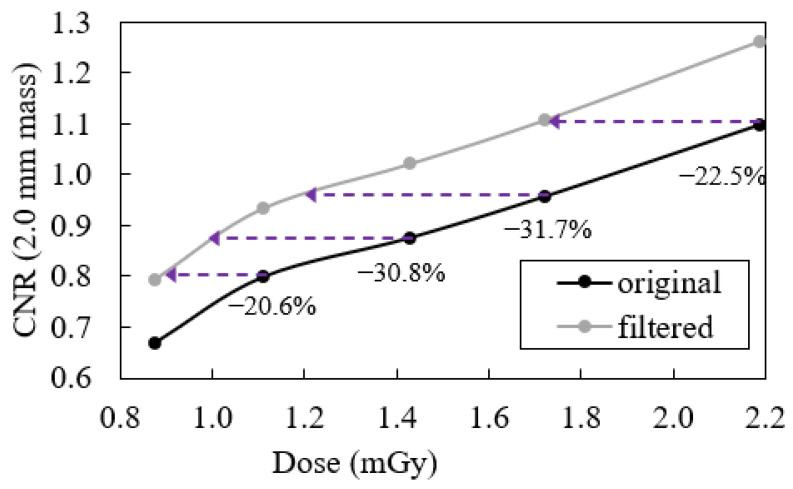
Values of CNR obtained for the original data and the filtered data of the Gammex 156 phantom as a function of dose. The purple arrows represents the possible dose reduction made by applying the filter to obtain the same CNR.

Figure 5 depicts the obtained CNR for the 2.0 mm mass as a function of the dose value for both the original and filtered data. The possible dose reductions made by applying the filter are also shown. It is possible to achieve a dose reduction whenever, after applying the filter, we achieve an identical CNR to that obtained with one of the original acquisitions.

**Figure 6:**
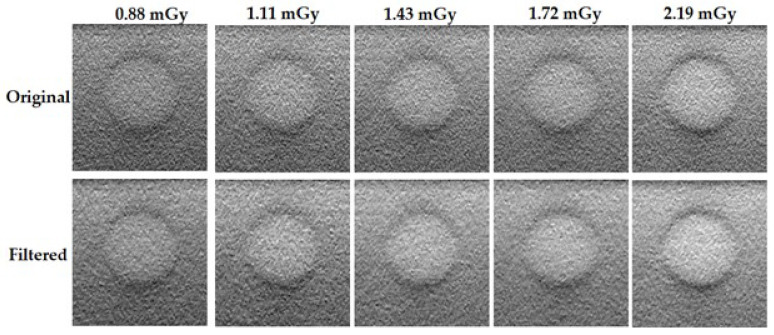
Images of a 2.0 mm mass of the Gammex 156 phantom obtained for the original (**up row**) and filtered data (**down row**) for each acquisition dose.

**Figure 7:**
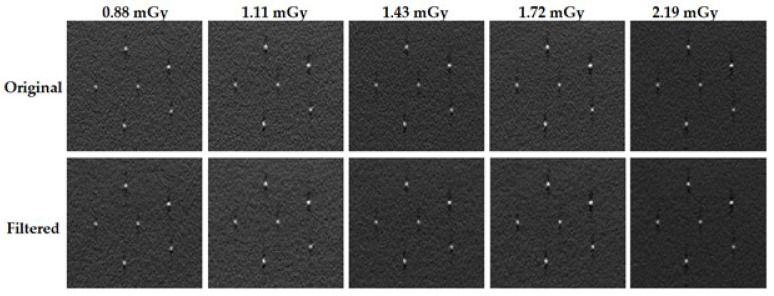
Images of a cluster of microcalcifications of the Gammex 156 phantom obtained for the original (**up row**) and filtered data (**down row**) for each acquisition dose.

For a visual inspection, images of the 2 mm lesion-like mass and a cluster of microcalcifications for each dose value are presented in Figure 6 and Figure 7, respectively. The first line of each figure concerns the original images, while the second line shows the images obtained after applying the filter with the respective optimal λ.

Table 3 is similar to Table 2 but for our phantom instead of Gammex 156. The optimal λ values obtained to achieve the minimization of the 2D TV values of the data are presented, as well as the TV values of the original and filtered data.

**Figure 8:**
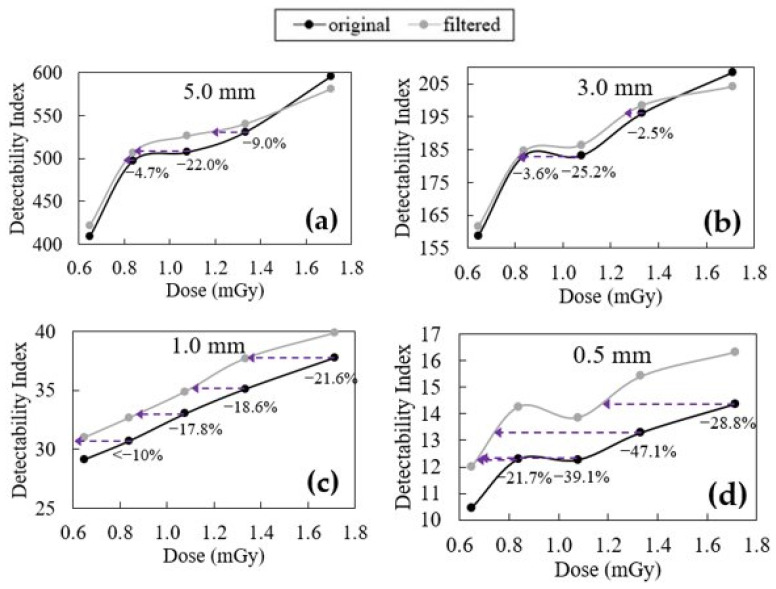
Detectability index values obtained for a circular signal with designer profile and diameters of (**a**) 5 mm, (**b**) 3 mm, (**c**) 1 mm, and (**d**) 0.5 mm for each acquisition dose of original and filtered data. The purple arrows represents the possible dose reduction made by applying the filter to obtain the same detectability index.

**Figure 9:**
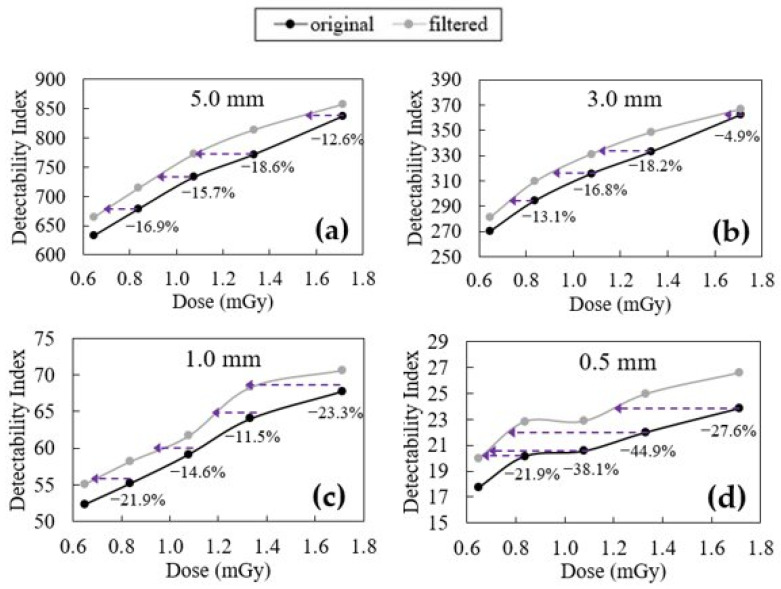
Detectability index values obtained for a circular signal with rectangular profile and diameters of (**a**) 5 mm, (**b**) 3 mm, (**c**) 1 mm, and (**d**) 0.5 mm for each acquisition dose of original and filtered data. The purple arrows represent possible dose reduction made by applying the filter to obtain the same detectability index.

In Figure 8 and Figure 9, the variations of the detectability index before and after the filter application as a function of the dose are presented for the two different configurations of $W_{task}$—designer profile and rectangular profile, respectively. Table 4 aims to complement the information derived from Figure 8 and Figure 9.

## 4. Discussion

In this work, the DBT data of two phantoms acquired with different dose values were reconstructed using manufacturer FBP and filtered using a straightforward 2D TV minimization filter. Our analysis encompassed the optimal λ value that minimizes the 2D TV for each phantom; the calculation of CNR and detectability index for original and filtered data, considering the several dose values for the acquisition.

Through the analysis of these results in Table 2, it is possible to verify that there exists a negative variation of the 2D TV value for every dose. This means that the 2D TV values diminish with the application of the filtering routine, indicating that this approach benefits image quality. The range of variation goes from 29.2% to 25.1%, decreasing as the dose increases, which is as expected because TV relates directly with the image noise. In addition, the higher the dose, the lower the noise in the image; therefore, the smaller the 2D TV and the lower its minimization. It is noticeable from these results that while all data (resulting from any dose) benefit from the filter application, images obtained with a lower dose (and therefore, with a lower quality) gain more with this approach. Actually, focusing only on the results obtained for the images with the lowest dose, it is possible to verify that the 2D value obtained after filtering is even smaller than the one obtained for the original images with the highest dose—analogous conclusions can be made for any other dose value that is below the maximum dose (2.19 mGy). These results suggest that the use of this filtering technique can be a step forward in achieving a better image quality without the need for applying high dose levels.

Generally, from Figure 5, it is possible to see that for each dose point, the application of the filtering routine allows for the CNR to be increased. Once again, these results indicate that this approach might contribute to a generalized increase in image quality for every dose value. Furthermore, it is also possible to check in the same figure how the filtering technique could impact on dose reduction. The purple arrows show how much the dose could be reduced with the application of the filtering technique to achieve the same CNR as the original images do. For example, the CNR obtained with a dose of 1.43 mGy could be obtained with a reduction of 30.8% in the dose if the filtering routine was applied. Similar results are obtained for the remaining dose levels: a dose reduction of 20.6%, 31.7%, and 22.5% could be obtained if the filter was applied to images obtained with doses of 1.11, 1.72, and 2.19 mGy, respectively. In this way, based on CNR values, one can assume that it is possible to acquire exams with lower doses (on average, about 26%) and to apply the filter to achieve the same quality of image that was obtained with higher doses.

By looking for the original images of both Figure 6 and Figure 7, it is noticeable that an increase in the dose values results in a global increase in image quality—there is an increase in the contrast and a clear diminish in the granularity present. When making a shift for the filtered images, especially for the ones of the lower doses, it is possible to see the same pattern that was seen for the original images when increasing the dose. The contrast increases, the boundaries of the lesions become more visible, and there is a generalized diminish in the noise of the image.

In Table 3, the variation of 2D TV in our phantom before and after the filter application ranged from −28.2% to −31.2%, also decreasing as the dose increases. These results show a larger variation than what was seen for Gammex 156 in Table 2, but they corroborate the idea that original images with lower doses benefit more from the application of the filtering routine. As it happened for the results in Table 2, the 2D TV value obtained for the filtered image with the lowest dose is lower than the 2D TV value for the highest dose original image—and analogous findings are made for the remaining dose levels. The fact these results are concordant is highly promising. For both phantoms, the filter application results in a diminishing of up to 30% in 2D TV value, and low-dose filtered 2D TV values are lower than high-dose original 2D values, which demonstrates that this approach is highly valuable and generalizable.

Starting with the designer profile (Figure 8), it can be seen that generally, the application of the filtering routine results in a better detectability for different dose levels and with different diameters of the profile. In fact, it is only the case for two points—the highest dose of the 5.0 mm and 3.0 mm profile—that the detectability index does not increase after the filter is applied.

As for the CNR, in Figure 8, with the purple arrows it is possible to quantify how much dose could be reduced to achieve the same detectability index if the filter is applied to the original images. For the configuration with a diameter of 5.0 mm, the smallest dose reduction is observed, being 4.7%, 22%, and 9.0% for the dose values of 0.84, 1.08, and 1.33 mGy. Similar conclusions can be driven for the configuration of 3.0 mm, where, for the same dose values, the dose decrease is 3.6%, 25.2%, and 2.5%. On the other hand, while for these two configurations the average dose decrease was, respectively, 11.9% and 10.4%, for the configurations of diameters 1.0 mm and 0.5 mm, it was 17.0% and 34.2%, respectively. As can be seen by the analysis of the average dose decrease and by the fact that in the 1.0 mm and 0.5 mm configurations each dose level benefits from at least a 10% decrease in dose after filtering, configurations with a smaller diameter gain more from the use of this filtering technique in terms of the detectability index improvement. This greater impact on the detectability index for minor lesions is very positive. It is expected that larger lesions are easier to detect in any condition than smaller lesions, which with lower doses (higher noise) can be obscured, limiting the possibility for dose decrease. Therefore, it is very important to ensure that these small lesions continue to be detected after filter application, even in low-dose environments.

When using the rectangular profile for the Wtask calculation (Figure 9), the results appear similar. However, in this configuration, all data points benefit from filtering when it comes to an increase in the detectability index. When considering dose reduction to achieve the same detectability index while using the filtering routine, the results are generally better than for those using the designer profile. For the configurations with 5.0, 3.0, 1.0, and 0.5 mm, the average dose reduction obtained with filtering is 16.0% (vs. 11.9% in the designer profile), 13.3% (vs. 10.4%), 17.8% (vs. 17%), and 33.1% (vs. 34.2%), respectively. As can be seen, in three out of the four different diameter sizes, the detectability indexes calculated considering a rectangular profile are higher than those with a designer profile. This does not happen only for the 0.5 mm configuration, where both profiles achieve their best results—both with an average dose reduction of above 30%. Despite this fact, both profiles present analogous results: configurations with smaller diameters tend to benefit more from the filtering routine application in terms of the increase in the detectability index.

In Table 4, it is possible to verify the variation (in percentage) of the detectability index after filtering for each configuration of the two profiles across the five dose values used. It can be seen that even though the highest increases in detectability index occur in the designer profile (13.9% for doses of 0.84 mGy and 1.33 mGy in the 0.5 mm configuration), the rectangular profile showed more consistent results. The average increases in the detectability index by dose in the rectangular profile are 6.20%, 6.68%, 6.00%, 6.98%, and 4.50%; for doses of 0.65 mGy, 0.84 mGy, 1.08 mGy, 1.33 mGy, and 1.71 mGy, respectively. On the other hand, for the same doses but for the designer profile, this increase takes values of 5.90%, 5.63%, 5.53%, 5.98%, and 3.2%, respectively. So, for every dose level, the rectangular profile achieves higher average increases in the detectability index. However, when analyzing all of the increases one by one, it is possible to verify that the designer profile showed higher detectability indexes than the rectangular profile in every dose level for configurations of 1.0 mm and 0.5 mm. In fact, both configurations agree on these results: the increase is much higher for configurations with smaller diameters than for the 5.0 mm and 3.0 mm (where for the designer profile there are two occurrences of detectability index decrease after filtering). This fact shows that while the detectability index generally increases when the filtering routine is applied, smaller configurations benefit more from this approach. This fact means that while the application of this filter might help to increase the detectability of lesions of different sizes, it is the detection of smaller lesions that will gain more from the use of this methodology. Summarizing, the use of this approach helps to increase the confidence in detecting lesions of all sizes while diminishing the changes of overlooking smaller lesions that could be harmful.

As a first approach for the study of the dose, this TV minimization algorithm was applied in-plane and not to the entire volume. In the future, the filter should be applied in 3D, and the image quality evaluation of 3D CNR or the detectability index should consider the z-direction. In addition, it is also important to consider the application of the filter to one of the most usual types of noise present in medical images, which is a combination between Gaussian and Poisson noise [35].

On the other hand, in this work, we use the detectability index based on the NPW matched filter model, as this model has shown strong correlation with observer data. Future studies may include the detectability index for several variants of an NPW matched filter observer model, such as the NPW observer model with an eye filter (NPWE).

## 5. Conclusions

An optimized digital filter for TV minimization in DBT imaging has been used to study the possibility of dose reduction through image quality improvement. This filter adopts a Poisson noise model and solves the minimization problem directly, with this being a fast and straightforward application. In addition, it is not a black box since the mathematical problem was solved by us for DBT data and we know all the basic principles behind it. This factor is important because it allows for greater adaptability to different conditions, knowing a priori the type of outcome that we will have.

This study has the advantage of analyzing real phantom images. Both the quantitative and qualitative analyses performed showed the relevance of this approach in improving the image quality in DBT, and thus making true and real the possibility of a dose reduction in DBT exams.

In addition, the filter implementation methodology and image analysis here described are highly reproducible, which is important for comparisons between different scanners and reconstruction algorithms.

## Figures and Tables

**Table 1:** Acquisition parameters for both phantoms.

	Gammex 156			Our Phantom	
**kVp**	**Exposure (mAs)**	**Dose (mGy)**	**kVp**	**Exposure (mAs)**	**Dose (mGy)**
30	56	0.88	28	56	0.65
	71	1.11		71	0.84
	90	1.43		90	1.08
	110	1.72		110	1.33
	140	2.19		140	1.71

**Table 2:** Two-dimensional TV values for the original data and filtered data of the Gammex 156 phantom obtained with the optimum λ values found for each dose. The variation in percentage between the original and filtered 2D TV values are also presented.

Gammex 156			2D TV	
**Dose (mGy)**	λ	**Original**	**Filtered**	**var**
0.88	156	8.59 × $10^{7}$	6.09 × $10^{7}$	−29.2%
1.11	149	7.73 × $10^{7}$	5.60 × $10^{7}$	−27.6%
1.43	166	7.20 × $10^{7}$	5.25 × $10^{7}$	−27.0%
1.72	230	6.50 × $10^{7}$	4.81 × $10^{7}$	−25.9%
2.19	286	6.41 × $10^{7}$	4.80 × $10^{7}$	−25.1%

**Table 3:** Two-dimensional TV values for the original data and filtered data of our phantom obtained with the optimum λ values found for each dose. The variation in percentage between the original and filtered 2D TV values are also presented.

Our Phantom			2D TV	
**Dose (mGy)**	λ	**Original**	**Filtered**	**var**
0.65	143	9.06 × $10^{7}$	6.24 × $10^{7}$	−31.2%
0.84	155	8.39 × $10^{7}$	5.79 × $10^{7}$	−30.9%
1.08	164	7.95 × $10^{7}$	5.55 × $10^{7}$	−30.2%
1.33	175	7.57 × $10^{7}$	5.35 × $10^{7}$	−29.4%
1.71	199	6.96 × $10^{7}$	4.99 × $10^{7}$	−28.2%

**Table 4:** Variation between the detectability index values of the original and filtered data.

Dose (mGy)	Designer Profile				Rectangular Profile			
	Ø 5.0 mm	Ø 3.0 mm	Ø 1.0 mm	Ø 0.5 mm	Ø 5.0 mm	Ø 3.0 mm	Ø 1.0 mm	Ø 0.5 mm
0.65	3.0%	1.8%	6.0%	12.8%	4.8%	4.0%	4.9%	11.1%
0.84	1.9%	0.7%	6.0%	13.9%	5.0%	4.9%	5.1%	11.7%
1.08	3.5%	1.7%	5.4%	11.5%	5.1%	4.7%	4.1%	10.1%
1.33	1.8%	1.3%	6.9%	13.9%	5.2%	4.4%	6.3%	12.0%
1.71	−2.5%	−2.1%	5.3%	12.1%	2.4%	1.2%	4.1%	10.4%

## Data Availability

The data is not available due to privacy restrictions.
